# Case report: primary osteonecrosis associated with thrombophilia-hypofibrinolysis and worsened by testosterone therapy

**DOI:** 10.1186/s12878-017-0076-x

**Published:** 2017-03-27

**Authors:** Michael Ian Jarman, Kevin Lee, Ariel Kanevsky, Sarah Min, Ilana Schlam, Chris Mahida, Ali Huda, Alexander Milgrom, Naila Goldenberg, Charles J. Glueck, Ping Wang

**Affiliations:** From the Jewish Hospital, Internal Medicine Resident Graduate Medical Education Department, 4777 East Galbraith Road, Cincinnati, OH 45236 USA

**Keywords:** Osteonecrosis, Thrombophilia, Hypofibrinolysis, Testosterone, Anticoagulation

## Abstract

**Background:**

Familial and acquired thrombophilia are often etiologic for idiopathic hip and jaw osteonecrosis (ON), and testosterone therapy (TT) can interact with thrombophilia, worsening ON.

**Case presentation:**

Case 1: A 62-year-old Caucasian male (previous deep venous thrombosis), on warfarin 1 year for atrial fibrillation (AF), had non-specific right hip-abdominal pain for 2 years. CT scan revealed bilateral femoral head ON without collapse. Coagulation studies revealed Factor V Leiden (FVL) heterozygosity, 4G/4G plasminogen activator inhibitor (PAI) homozygosity, high anti-cardiolipin (ACLA) IgM antibodies, and endothelial nitric oxide (NO) synthase (eNOS) T786C homozygosity (reduced conversion of L-arginine to NO, required for bone health). Apixaban 5 mg twice daily was substituted for warfarin; and L-arginine 9 g/day was started to increase NO. On Apixaban for 8 months, he became asymptomatic. *Case 2:* A 32-year-old hypogonadal Caucasian male had 10 years of unexplained tooth loss, progressing to primary jaw ON with cavitation 8 months after starting TT gel 50 mg/day. Coagulation studies revealed FVL heterozygosity, PAI 4G/4G homozygosity, and the lupus anticoagulant. TT was discontinued. Jaw pain was sharply reduced within 2 months.

**Conclusions:**

Idiopathic ON, often caused by thrombophilia-hypofibrinolysis, is worsened by TT, and its progression may be slowed or stopped by discontinuation of TT and, thereafter, anticoagulation. Recognition of thrombophilia-hypofibrinolysis before joint collapse facilitates anticoagulation which may stop ON, preserving joints.

## Background

Osteonecrosis (ON) is often secondary to high-dose, long-term steroids or alcoholism, with primary (idiopathic) ON defined when known secondary etiologies are ruled-out [1]. The development of primary ON appears to follow a sequence of events initiated by familial or acquired thrombophilia [2–4], venous occlusion causing osseous venous outflow obstruction, leading to increased intraosseous venous pressure, reduced arterial flow, ischemia, bone infarction and eventual joint collapse [3–7]. Venous occlusion is the initiating event in experimental models of ON [8], and enoxaparin can prevent steroid induced ON [9]. Heritable or acquired thrombophilia-hypofibrinolysis alone, or augmented by testosterone therapy (TT) [9, 10] or clomiphene given to raise testosterone (in men) [10] are thought to lead to thrombotic venous occlusion and thence to osteonecrosis [3, 5–7]. In the current report, our specific aim was to describe the association of familial thrombophilia (Factor V Leiden heterozygosity), acquired thrombophilia (lupus anticoagulant, high anticardiolipin [ACLA] antibody IgM), and hypofibrinolysis (4G4G homozygosity for the plasminogen activator inhibitor −1 mutation) with hip and jaw ON, and the interaction of TT with familial thrombophilia in ON.

## Methods

Studies were carried out following a protocol approved by the Institutional Review Board with signed informed consent.

### Cases with idiopathic osteonecrosis and Controls

To provide a frame of reference for the two cases in the current report, thrombophilia-hypofibrinolysis measures in 240 cases with idiopathic osteonecrosis sequentially referred to our center for coagulation studies are provided, along with comparisons to 110 previously reported healthy, normal controls [11] (48 men, 62 women). Controls were selected from healthy hospital employees without osteonecrosis and without chronic disease states.

### Measures of thrombophilia and hypofibrinolysis

PCR measures of the Factor V Leiden, Prothrombin, methylenetetrahydrofolate reductase (MTHFR), and plasminogen activator inhibitor (PAI-1 gene) mutations were done along with serologic measures of Lp (a), homocysteine, factors VIII and XI, antigenic proteins C, total S, free S, and antithrombin III, lupus anticoagulant, and anticardiolipin antibodies (ACLA) IgG and IgM, using previously reported methods [11, 12]. Serologic tests were done before anticoagulation. Plasminogen activator inhibitor-1 activity was not measured.

### Statistical methods

The data were processed by SAS 9.4. Comparison of measures of thrombophilia-hypofibrinolysis between cases and controls was done by Chi-Square analysis, excepting those comparisons where the expected cell size was <5, when Fisher’s exact *X* ^2^ tests were used.

## Results

### Case presentation

#### Case 1

A 62-year-old Caucasian male had a past history of deep venous thrombosis (DVT) in the right lower extremity (1995, after cervical laminectomy), and superficial venous thrombosis of the right greater saphenous vein (2003, after ablation procedure). He was receiving warfarin for 1 year for paroxysmal atrial fibrillation (AF) (status-post ablation and failed cardioversion). He was evaluated for abdominal-flank-hip pain progressing over a 2-year period. An abdominal CT scan revealed previously undiagnosed ON of both femoral heads without collapse (Ficat [13] stage I). In absence of alcoholism, high-dose long-term corticosteroid therapy, or any other causes of secondary ON [6], the ON was thought to be primary-idiopathic. Coagulation studies revealed thrombophilic Factor V Leiden (FVL) heterozygosity, hypofibrinolytic 4G/4G homozygosity for the PAI-1 gene, thrombophilic elevated ACLA IgM (24 MPL, mid positive 20–80 MPL) and eNOS T786C homozygosity associated with reduced conversion of L-arginine to nitric oxide (NO), required for normal bone health [14]. The initial high ACLA IgM remained high on repeated testing. There were no other features of the antiphospholipid syndrome. Family screening revealed that both daughters were heterozygous for the FVL mutation, and one was homozygous for the PAI-1 4G/4G mutation. Because of difficulty maintaining an INR target of 2.5, Apixaban 5 mg twice daily was substituted for warfarin. L-arginine, 9 g/day, was given to increase NO production [15]. After 8 months on Apixaban, he was asymptomatic, but repeat imaging of the hips has not been done. We speculated that the warfarin, given for 1 year because of AF, may have concurrently stabilized his Ficat stage I ON.

#### Case 2

A 32-year-old hypogonadal Caucasian male had a 10-year history of unexplained progressive tooth erosion and tooth loss. Tooth loss accelerated 8-months after starting clomiphene (50 mg/day) to raise serum total and free testosterone, and a CT scan revealed cavitating ON of both jaws. The patient was referred to our center by his dental surgeon for evaluation. There were no etiologic factors for secondary ON. There was no evidence for excessive stomach acid or trauma (including teeth-grinding) related to tooth erosion, and no alcohol abuse, high-dose/long-term corticosteroids, or other secondary causes of ON. He had never received bisphosphonates, associated with jaw ON [16]. Coagulation studies revealed heterozygosity for the FVL mutation, homozygosity for the PAI-1 4G/4G mutation, and the lupus anticoagulant was positive, and remained positive on repeat testing.

TT was discontinued, with a sharp reduction in jaw pain within 2 months.

To provide a frame of reference to the two patients of the current report, Table 1 [17] displays comparisons of coagulation measures in 240 patients with primary ON versus those in 110 healthy normal controls. Over a period of 24 years beginning in 1992, we assessed 372 patients being referred for a new radiologic diagnosis of ON of either the hip or knee [17, 18] of whom 240 had primary idiopathic ON (not secondary to high dose-long term corticosteroids, alcoholism, sickle cell disease, dislocation, etc.). The 240 patients were compared against healthy normal controls (*n* = 110), except in the comparison of eNOS [14] mutations for which only 72 historical controls were evaluated. Due to diagnostic lab testing limitations, not every control or patient was able to be evaluated for every form of thrombophilia or hypofibrinolysis. These results allowed us to estimate the prevalence of thrombophilia and hypofibrinolysis in the healthy controls without ON, and amongst patients with primary ON, Table 1.

**Table 1:** Coagulation abnormalities in 240 patients with primary osteonecrosis, compared with 110 healthy normal controls

Coagulation measures	Abnormal range	Primary ON (*n* = 240)	Normal controls (*n* = 110)	Case vs control p ^a^
Factor V Leiden	TC, TT	21/235 (9%)	2/109 (2%)	.014
Prothrombin gene	TC, TT	9/228 (4%)	3/110 (3%)	.76 (F)
MTHFR	TT	46/226 (20%)	32/109 (29%)	.068
PAI-1 gene	4G4G	66/225 (29%)	26/104 (25%)	.42
Homocysteine	Dated cut point^b^	32/212 (15%)	5/107 (5%)	.0061
ACLA IgG	Dated cut point^c^	11/210 (5%)	6/109 (6%)	.92
ACLA IgM	Dated cut point^d^	27/209 (13%)	2/109 (2%)	.0011
Lupus anticoagulant	positive	2/203 (1%)	2/110 (2%)	.61 (F)
Lp(a)	≥35 mg/dl	61/205 (30%)	21/107 (20%)	.054
Factor VIII	>150%	47/175 (27%)	7/103 (7%)	<.0001
Factor XI	>150%	15/169 (9%)	3/101 (3%)	.060
Protein C	<73%	10/204 (5%)	6/96 (6%)	.63
Protein S	<63%	1/206 (0.5%)	4/96 (4%)	.037 (F)
Free S	<66%	8/186 (4%)	2/96 (2%)	.50 (F)
Antithrombin III	<80	8/203 (4%)	2/96 (2%)	.51 (F)
			Compare with 72 controls	
eNOS	TC, TT	90/138 (65%)	28/72 (29%)	.0003

^a^comparisons made by Chi-square test, unless there were cells which had expected counts <5 in 2 × 2 table where Fisher’s exact test (F) was used

^b^dated cut point for Homocysteine high: ≥15 (11/15/08-12/2/14); ≥10.4 (after 12/3/14)

^c^ dated cut point for IgG high: ≥23 GPL (before 10/31/12); ≥15 (after 11/1/12)

^d^ dated cut point for IgM high: ≥10 MPL (before 4/30/12); ≥13 (after 5/1/12)

The two patients of our current report were heterozygous for the FVL mutation, one had elevated ACLA, and one was homozygous for the eNOS T786C mutation [14]. Our 240 patients with primary ON differed from normal controls by having FVL heterozygosity (like the 2 patients in the current report), high homocysteine, high ACLA IgM, high Factor VIII, and hetero-homozygosity for the eNOS T786C mutation, Table 1.

## Discussion

Arthralgia of the hips [19] and knees [20] is a very common complaint in the outpatient setting, often requiring diagnostic imaging studies [21–23]. Primary ON [6] is not a common cause of arthralgia [24], and the diagnosis and therapy of ON remains poorly understood by many clinicians. While the prevalence of early-stage (pre-joint collapse) primary ON appears to be relatively low [25], it is important to diagnose, because, as we have shown, treatment with long-term anticoagulation in patients with familial or acquired thrombophilia-hypofibrinolysis and with primary ON (Ficat [13] stage I-II, pre-joint collapse) often results in complete symptomatic relief and long-term joint preservation of both knees [7] and hips [26], and may ameliorate osteonecrosis of the jaw [27]. The major clinical barrier to reaching this therapeutic benefit is the lack of awareness of the association between thrombophilia- hypofibrinolysis with primary ON [1, 3, 14, 28–31] which we are addressing in the current report.

There appear to be etiologic associations between factor V Leiden heterozygosity [3, 28, 32], hyperhomocysteinemia [33], high ACLA IgM [34], high Factor VIII [35], hetero-homozygosity for the T786C eNOS mutation [14] and primary ON. ON, particularly multifocal, occurs in patients with the antiphospholipid antibody syndrome [36]. Exogenous testosterone therapy (TT) in patients with familial or acquired thrombophilia-hypofibrinolysis promotes development of ON [32, 37] of the femoral head [32] and jaw [10].

In patients with early stage primary-idiopathic ON, before segmental collapse of hips or knees has occurred (Ficat [13] stage I or II), with a heritable thrombophilia or hypofibrinolysis, anticoagulation therapy of at least 1 year has been shown to arrest progression of ON and lead to clinically significant pain relief [7, 26, 38, 39]. We have previously [26] reported that long term anticoagulation (4 to 16 years) stops progression of idiopathic hip ON associated with familial thrombophilia (5 patients with Factor V Leiden Heterozygotes and 1 patient with resistance to activated protein C). On 4–16 years anticoagulation, 9 hips in these 6 patients, 8 originally Ficat II, 1 Ficat 1 remained unchanged [26] in contrast to untreated ON Ficat stage II where 50–80% of hips progress to collapse (Ficat III-IV) within 2 years of diagnosis [40, 41]. Left untreated, ON of the hip inevitably leads to irreversible segmental or total joint collapse [41] (i.e., Ficat III- IV) requiring joint replacement, typically within 2 years of initial diagnosis [40–45].

There were no clinically significant bleeding episodes [26]. Long term anticoagulation initiated in Ficat I II idiopathic hip in patients heterozygous for the Factor V Leiden mutation may change the natural history of the disease [26].

Long term anticoagulation is also effective in thrombophilic patients with early (pre-collapse) primary ON of the knee [7]. In 6 patients with knee osteonecrosis, all 6 with thrombophilia, 4 with concurrent hypofibrinolysis, we determined prospectively whether anticoagulation with Enoxaparin could prevent collapse, progression to osteoarthritis, ameliorate pain, and restore function [7]. The 6 patients were treated with Enoxaparin (40–60 mg/day for ≥ 3 months) as mandated by an FDA-approved protocol. In post-Enoxaparin prospective follow-up, patients were reassessed clinically every 4–6 months and X-rayed every year. The 6 patients had follow-up for 15.1, 7.5, 3.9, 2.25, 2, and 1 years [7]. None progressed to joint collapse or severe osteoarthritis; 4 became and remained asymptomatic at 2, 3.9, 7.5, and 15.1 year follow-up [7]. Thrombophilic-hypofibrinoytic patients with knee ON treated with Enoxaparin have had no collapse or progression to severe osteoarthritis; and most have had resolution of pain and restoration of full function [7].

In the jaw, when left untreated, ON leads to progressive tooth loss with failure to heal, jaw bone cavitation, and chronic pain syndromes [10, 16]. Paralleling the patient in our current report, we have previously reported a very similar case of primary ON of the jaw in a 55 year old Caucasian man [10]. He also had FVL heterozygosity, and rapid progression of disease 6 months after starting TT gel 50 mg/day, with development of high serum T (963 ng/dl, laboratory upper limit [46] 800 ng/dl) and high estradiol (50 pg/ml, laboratory UNL 42.6 pg/ml). As in our current patient, the development of jaw ON appeared soon (6 months) after initiation of TT therapy [10], which then interacted with the patient’s familial and acquired thrombophilia and familial hypofibrinolysis, promoting and worsening the jaw ON [18, 32]. We have previously reported in a pilot study, that warfarin therapy in patients with both Factor V Leiden heterozygosity and osteonecrosis of the jaw was effective in reducing jaw pain [27].

In patients with thrombophilia-hypofibrinolysis and thrombotic events on TT, continuation of TT, even with adequate concurrent anticoagulation, leads to repetitive thrombotic events [47].

When the thrombus-promoting TT therapy is stopped, and anticoagulation started, we speculate that venous outflow is restored, increased venous pressure in the bone is reduced, facilitating increased arterial flow, reducing osseous ischemia [7, 26]. This is accompanied, as in our current case of jaw ON, by reduction of symptoms, and, in longer term anticoagulation studies, by stopping and reversing osteonecrosis [7, 26]. It is relevant that experimental models of ON have shown venous occlusion to be the initiating event [8, 48], and that treatment with enoxaparin in experimental animals has the potential to prevent steroid-associated ON [48]. Giving TT to mice hetero-and homozygous for the Factor V Leiden mutation [49, 50] and also having experimental antiphospholipid syndrome [50], and using animal models of venous occlusion and ON [8, 48] would allow basic science studies of the relationship of TT and thrombophilia to ON [18].

Historically, in adults with primary ON of weight bearing joints, the most common treatments include invasive forms of secondary and tertiary prevention, including core decompression [51] with or without stem cell infusion [52], vascularized fibular graft [53], and ultimately total joint replacement [54]. Hence, new diagnoses of ON, initially made by the radiologist and confirmed by the orthopedic or dental surgeon or clinician [55], rarely undergo a rigorous workup for thrombophilia or hypofibrinolysis [5]. This represents a clinically important missed-opportunity as many patients may be inadequately treated for their joint pain [56] while thrombophilia-hypofibrinolysis, a treatable [7, 26] causative etiology of primary ON (Ficat Stages I-II), goes unaddressed [5].

## Conclusion

Primary ON is often caused by underlying familial and acquired thrombophilia and hypofibrinolysis, and can be worsened by TT or testosterone-elevating clomiphene. In patients with primary ON, it is important for diagnostic and therapeutic reasons to determine whether thrombophilia-hypofibrinolysis are present, and whether TT is being used. To stop progression of early primary ON at Ficat Stage I or II before joint collapse in patients with thrombophilia-hypofibrinolysis, discontinuing TT is essential and may slow or stop progression of the ON. Initiating anticoagulation may stop progression of ON in thrombophilic patients without joint collapse and without progression to jaw cavitation, thus, avoiding the usual natural history of untreated ON, which is total joint replacement within 2 years of initial diagnosis, and in the jaw, chronic pain syndrome with jaw cavitation and recurrent supra-infection.

## Abbreviations

ACLA: Anti-cardiolipin IgM antibodies
AF: Atrial fibrillation
DVT: Deep venous thrombosis
eNOS: endothelial nitric oxide synthase
FVL: Factor V Leiden
MTHFR: Methylenetetrahydrofolate reductase
NO: Nitric oxide
ON: Osteonecrosis
PAI: Plasminogen activator inhibitor
TT: Testosterone therapy
